# Benzylpenicillin concentrations in umbilical cord blood and plasma of premature neonates following intrapartum doses for group B streptococcal prophylaxis

**DOI:** 10.1186/s40748-023-00163-3

**Published:** 2023-07-01

**Authors:** Amadou Samb, Thomas H. Dierikx, Yuma A. Bijleveld, Timo R. de Haan, Caspar J. Hodiamont, Elisabeth van Leeuwen, Anton H. L. C. van Kaam, Ron A. A. Mathôt, Douwe H. Visser

**Affiliations:** 1 Department of Pharmacy and Clinical Pharmacology, Amsterdam UMC Location University of Amsterdam, Meibergdreef 9, Amsterdam, the Netherlands; 2 Amsterdam Reproduction & Development, Amsterdam, the Netherlands; 3 Department of Pediatric Gastroenterology, Amsterdam UMC Location Vrije Universiteit Amsterdam, Boelelaan 1117, Amsterdam, the Netherlands; 4 Amsterdam Gastroenterology Endocrinology Metabolism, Amsterdam, the Netherlands; 5 Department of Neonatology, Amsterdam UMC Location University of Amsterdam, Meibergdreef 9, Amsterdam, the Netherlands; 6 Department of Medical Microbiology and Infection Prevention, Amsterdam UMC Location University of Amsterdam, Meibergdreef 9, Amsterdam, the Netherlands; 7 Department of Obstetrics, Amsterdam UMC Location University of Amsterdam, Meibergdreef 9, Amsterdam, the Netherlands

**Keywords:** GBS prophylaxis, Benzylpenicillin, Pharmacology

## Abstract

**Background and method:**

Dutch obstetrics guideline suggest an initial maternal benzylpenicillin dose of 2,000,000 IU followed by 1,000,000 IU every 4 h for group-B-streptococci (GBS) prophylaxis. The objective of this study was to evaluate whether concentrations of benzylpenicillin reached concentrations above the minimal inhibitory concentrations (MIC) in umbilical cord blood (UCB) and neonatal plasma following the Dutch guideline.

**Results:**

Forty-six neonates were included. A total of 46 UCB samples and 18 neonatal plasma samples were available for analysis. Nineteen neonates had mothers that received intrapartum benzylpenicillin. Benzylpenicillin in UCB corresponded to concentrations in plasma drawn directly postpartum (R2 = 0.88, *p* < 0.01). A log-linear regression suggested that benzylpenicillin concentrations in neonates remained above the MIC threshold 0.125 mg/L up to 13.0 h after the last intrapartum dose.

**Conclusions:**

Dutch intrapartum benzylpenicillin doses result in neonatal concentrations above the MIC of GBS.

## Background

Early-onset neonatal sepsis (EOS) is a severe infectious disease with signs of bacteremia resulting from vertical transmission of pathogens originating in the maternal genital tract or amniotic fluid [1]. EOS is contracted before or during labor and typically presents with signs and symptoms within the first 72 h of life. It is associated with both a high neonatal mortality and morbidity [2]. Group B streptococcus (GBS), S*treptococcus agalactiae*, is one of the leading pathogens for EOS and 10–30% of pregnant women carry GBS in the genital tract, allowing for vertical transmission during delivery [3].

In the last 30 years the incidence of GBS positive EOS has declined drastically from 1.7 to 0.4 cases per 1,000 live births due to the implementation of widespread and systematic screening for maternal risk factors for EOS, such as GBS colonization and/or chorioamnionitis. Some countries have implemented a screen all strategy around 35–37 weeks’ gestation, and give prophylaxis to all screen positives while other countries follow a risk based strategy, where screening only takes place for instance in preterm delivery or prolonged rupture of membranes. In the Netherlands, if risk factors are present mothers are treated with targeted intrapartum antibiotic prophylaxis until delivery [3, 4]. Maternal intravenous benzylpenicillin prophylaxis within at least 4 h before delivery is considered adequate for the prevention of EOS. Benzylpenicillin concentrations assessed in fetal serum and amniotic fluid typically exceed the minimal inhibitory concentrations (MIC) of GBS at delivery [3, 5].

Several studies have evaluated benzylpenicillin concentrations in umbilical cord blood (UCB) and fetal blood in term pregnancies with maternal benzylpenicillin loading doses of 5,000,000 IU, followed by 2,500,000 IU every 4 h intrapartum [4–6]. However, the current Dutch obstetric guideline advices lower doses, i.e. a loading dose of 2,000,000 IU followed by 1,000,000 IU every 4 h [7], but evidence for the latter dose is lacking and it is unknown whether this results in adequate benzylpenicillin concentrations in the neonate. Furthermore, the magnitude of fetal exposure to benzylpenicillin following intrapartum prophylaxis before 37 weeks’ gestation is unknown. This evidence is needed because trimester-dependent changes in maternal body composition, placental function and fetal organ maturation may result in different pharmacokinetic (PK) properties [8].

The primary aim of this study was to assess whether maternal intrapartum intravenously administered benzylpenicillin at doses according to the current Dutch obstetric guideline is sufficient to produce adequate therapeutic benzylpenicillin concentrations in neonates. As a secondary objective, additional intrapartum antibiotic (amoxicillin, cefazolin and gentamicin) concentrations in UCB were investigated.

## Methods

### Study design and population

Neonatal plasma and UCB samples collected as part of a previous multicenter prospective observational cohort study were used (*The Diagnostic Accuracy of Presepsin in Early-Onset Neonatal Sepsis: a Prospective Cohort Study, submitted*). That study was conducted between August 2018 and June 2021. Neonates admitted to the neonatology ward of the Amsterdam UMC (Amsterdam, the Netherlands) or OLVG East and West (Amsterdam, the Netherlands), undergoing sepsis work up and receiving antibiotics according to the Dutch early onset neonatal sepsis guideline [7] were eligible for inclusion. Infants were excluded in case of a confirmed congenital infection (toxoplasmosis, rubella, cytomegalovirus infection, syphilis and herpes). For the current study documented maternal intrapartum antibiotic treatment was an additional entry criterion for inclusion and analysis.

The original study, as well as the amendment for the current study was approved by the medical ethics committee (WO 18.020). Written and informed consent was required from both parents or legal guardians for study participation and use of blood samples and clinical data.

### Data collection

Immediately after delivery, 1 mL UCB was collected in EDTA tubes, which was assumed to represent fetal blood. If possible, 0.2 mL neonatal plasma was drawn during initial sepsis work up directly postpartum before initiation of neonatal antibiotic treatment. All samples were centrifuged at 2,000 × g within 4 h after collection and the plasma was stored at -80 °C until analysis.

Benzylpenicillin, amoxicillin, gentamicin and cefazolin concentrations were measured in UCB and plasma samples using tandem liquid chromatography-mass spectrometry (LC–MS/MS). The accuracy was acceptable at all quality control (QC) levels (acceptance for QC_low_: 80%—120%; QC_middle_ & QC_high_: 85%—115%) for benzylpenicillin, amoxicillin, gentamicin and cefazolin with ranges between 98.3%—101.1%, 97.5%—99.3%, 96.6%—118% and 96.3%—110.0%, respectively. Within-day- and between-day imprecision were below the acceptance criteria of 15%.

Additional clinical, demographic and anthropomorphic data were collected from the digital medical patient files (EPIC, Verona, Wisconsin, USA). The following maternal data were collected: benzylpenicillin, cefazolin, gentamicin and amoxicillin maintenance dose (2,000 mg, 5 mg/kg and 1,000 mg respectively), time of dose, weight, length, presence of chorioamnionitis, gestational age at delivery, concomitant medication, GBS colonization status, multiple pregnancy, gravidity and parity. Neonatal data collected were: Time of dose, concomitant medication, birth weight, gestational age, sex, presence of perinatal asphyxia.

### Statistical analysis

Descriptive statistics, data handling and data visualization were performed with R [9]. If the measured UCB concentrations of the antibiotics were not normally distributed, median concentrations and interquartile range (IQR) were reported. Correlation between benzylpenicillin in UCB and neonatal plasma was tested to assess interchangeability. Tests of correlation as well as log-linear regression methods were performed to relate intrapartum benzylpenicillin dosing times to UCB benzylpenicillin concentrations. This relationship was then used to evaluate MIC target attainment. The target MIC used for benzylpenicillin was 0.125 mg/L, which is the highest MIC in the wild type distribution and the clinical breakpoint of the European Committee on Antimicrobial Susceptibility Testing (EUCAST) for GBS meningitis [10, 11]. For cefazolin and amoxicillin the EUCAST clinical breakpoint MICs for *E. coli* and GBS, two frequently isolated pathogens in EOS, were used. If no clinical breakpoint was defined by EUCAST, the highest MIC in the wild type distribution was used as target MIC [10]. For *E. coli*, the target MICs used for cefazolin and amoxicillin were 4 mg/L and 8 mg/L, respectively [11]. For GBS, the target MIC of amoxicillin was 0.125 mg/L, which is the highest MIC in the wild type distribution [10] For GBS, there is no clinical breakpoint for cefazolin, nor is there a known wild type distribution in the EUCAST database of antimicrobial wild type distributions. No target MICs for gentamicin were included, since gentamicin effect is dependent on maximum concentrations (C_max_), which is not represented by the measured drug concentrations. The two-tailed level of acceptance for the rejection of the null-hypotheses was *p* ≤ 0.05.

## Results

A total of 333 neonates were included in the study between July 30^th^ 2018 and July 23^rd^ 2021 of whom 46 mothers were treated with intrapartum antibiotics and UCB material was available. Nineteen of these mothers received intrapartum EOS prophylaxis with benzylpenicillin in a dose of 2,000,000 IU followed by 1,000,000 IU every 4 h. The demographic characteristics of the included infants and their mothers have been depicted in Table 1. The median gestational age for the benzylpenicillin group was 29.3 weeks. All infants were premature, except for one (GA = 42 weeks). The median maternal age during delivery was 30.2 years. Nine neonatal plasma samples drawn directly postpartum were available for comparison to UCB benzylpenicillin concentrations (Table 1).

**Table 1:** Basic characteristics of the study population stratified for benzylpenicillin and other antibiotics

Intrapartum antibiotic type	**Benzylpenicillin**	**Other antibiotics** ^a^
Number of infants	19	27
Female infants n (%)	10 (52.6)	15 (55.6)
Maternal age in years (median (IQR))	30.2 (28.0—33.0)	32.5 (29.0—35.0)
**Mode of delivery (n)**		
Vaginal n(%)	18 (94.7)	9 (33.3)
Caesarian n(%)	1 (5.3)	18 (66.7)
PROM duration in hours (median (IQR))	3.5 (0—47.3)	12.5 (0—74.8)
Maternal GBS colonization		
Positive n (%)	6 (31.6)	4 (14.8)
Negative n (%)	13 (68.4)	10 (37.0)
Unknown n (%)	-	13 (48.2)
Multiparous pregnancies n (%)	4 (21.1)	3 (11.1)
Gestational age in weeks (median (IQR))	29.3 (27.1—30.8)	31.0 (28.2—36.0)
Birth weight in kg (median (IQR)	1.15 (0.93—1.67)	1.52 (1.19—2.56)
Umbilical cord blood samples (n)	19	27
Neonatal plasma samples (n)	9	9

*PROM* Premature Rupture Of Membranes, *GBS* Group B streptococcus; ^a^Other antibiotics are: amoxicillin (*n* = 14), gentamicin (*n* = 3) and cefazolin (*n* = 13)

**Fig. 1:**
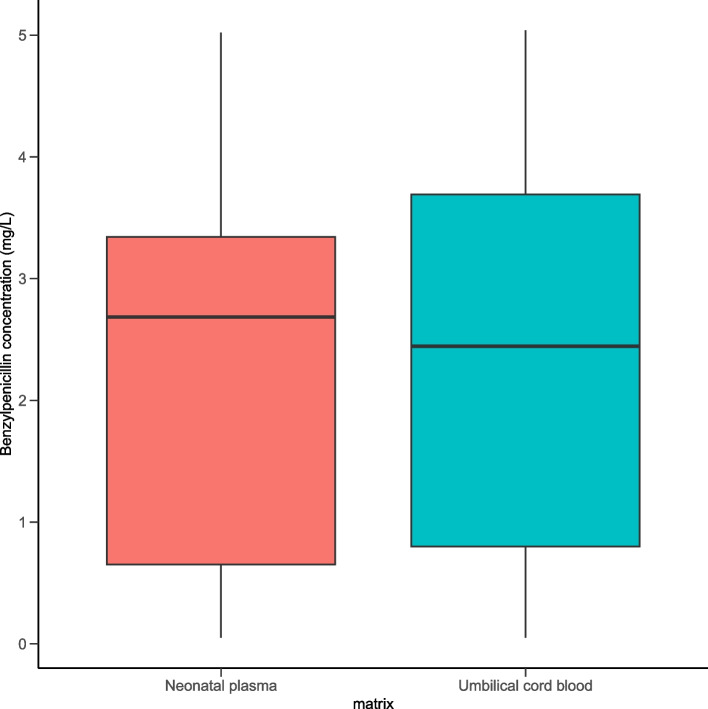
Boxplots of benzylpenicillin concentrations (mg/L) in umbilical cord blood (UCB) and neonatal plasma at birth (*N* = 9). The left boxplot depicts neonatal plasma concentrations. The right boxplot depicts UCB concentrations

**Fig. 2:**
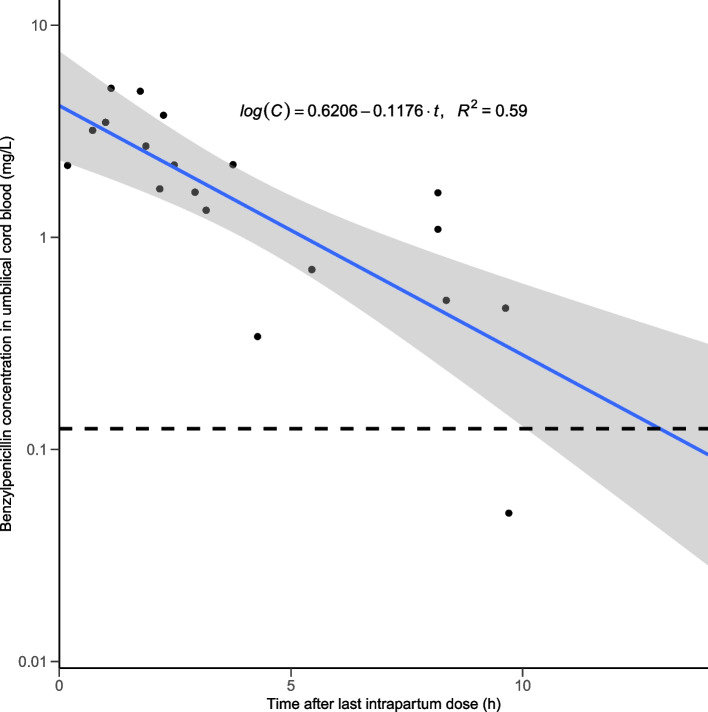
Benzylpenicillin concentrations in umbilical cord blood (mg/L) versus time after last intrapartum dose (hours). Points are measured concentrations. The blue line depicts the log-linear regression of the benzylpenicillin concentrations versus time after last intrapartum dose. The shaded area displays the 95% confidence interval of the regression line. The dashed horizontal line depicts the MIC threshold of 0.125 mg/L

The median concentration of benzylpenicillin in UCB was 1.69 (0.90 – 2.94) mg/L. Median concentrations in UCB and neonatal plasma at birth were similar (Fig. 1) and there was a strong correlation between benzylpenicillin concentrations in UCB and neonatal plasma at birth (R^2^ = 0.88, *p* < 0.01), indicating that UCB concentrations could be interpreted as fetal or neonatal concentrations. As expected, benzylpenicillin concentrations in UCB were dependent on the time after last intrapartum administration (Fig. 2). A clear decrease in UCB benzylpenicillin concentration is present. All but one observation were above the MIC of 0.125 mg/L for GBS, well past the 4 h dose interval. There was a significant log-linear relation (*p* < 0.01) between benzylpenicillin concentration and time after dose, indicating a moderate correlation (R^2^ = 0.59, Fig. 2). Based on the linear regression line, benzylpenicillin concentrations in UCB were above the MIC threshold of 0.125 mg/L for 13.0 h after the last intrapartum dose. When also taking the 95% confidence interval of the regression line into account, it seems more likely that concentrations reach subtherapeutic concentrations after 10 h. The slope of the regression line can be interpreted as an elimination constant k_e_, thus the elimination half-life can be calculated as ${t}_\frac{1}{2}=\frac{\mathrm{log}\left(2\right)}{0.1176}=2.6\mathrm{ hours}$. No covariate effect of GA on the relation between time after last intrapartum dose and benzylpenicillin concentration was found when included as an additional independent variable in the log-linear regression, since the slope of GA was not significant (*p* = 0.7).

The concentrations of amoxicillin, gentamicin and cefazolin were also measured in UCB. For amoxicillin the median concentration (*n* = 14) was 4.61 (2.53—6.32) mg/L, well above the GBS MIC threshold of 0.125 mg/L, though below the *E. coli* MIC of 8 mg/L. Cefazolin concentrations (*n* = 13) were also above the *E. coli* MIC threshold of 4 mg/L, with a median concentration of 17.5 (6.4—23.2) mg/L. Only 3 gentamicin concentrations were measured (below the lower limit of quantification, 1.33 mg/L and 3.50 mg/L).

## Discussion

UCB concentrations of benzylpenicillin, which is frequently used for prophylaxis in GBS-positive pregnancies, were above the suggested MIC threshold of 0.125 mg/L [11]. Although the internationally suggested dose of 5,000,000 IU followed by 2,500,000 IU every 4 h results in satisfactory fetal exposure [4, 5], this also seems the case for the lower doses of 2,000,000 IU, followed by 1,000,000 IU every 4 h, as suggested by the Dutch obstetric guideline [7]. As deducted by linear regression, benzylpenicillin concentrations in UCB remained above the MIC threshold of 0.125 mg/L up to 13.0 h after the last intrapartum dose. No relationship between GA and benzylpenicillin concentrations in UCB was found, though it is likely that data were insufficient for the detection of such a relationship. Furthermore, 18 of the 19 neonates that were exposed to intrapartum benzylpenicillin were premature with a GA range of 25 – 32 weeks, and the single term neonate had could be potentially seen as an outlier, with a GA of 42. Though it was evaluated whether exclusion of the patient of 42 weeks impacted the concentration–time profile, the slope of the log-linear regression did not change by a large margin. Moreover, exclusion of the patient of 42 weeks is unjustified, since it is part of the true population for GBS prophylaxis. Nonetheless these results mostly apply to the specific age group of premature neonates of GA 25—32 weeks. Thisis acceptable, since prematurity is a major risk factor that is considered for GBS prophylaxis. Benzylpenicillin T_1/2_ in UCB was 2.6 h, which is shorter than the reported T_1/2_ in term and preterm neonates (approximately 3.5 h) [12]. This could be the result of a shared clearance mechanism with the mother, since benzylpenicillin T_1/2_ is only 30 min in adults [13].

As the Dutch recommended benzylpenicillin dosing regimen results in therapeutic UCB concentrations, the international community may consider lower benzylpenicillin dose prophylaxis. Benzylpenicillin is a time-dependent antimicrobial and the bactericidal effect is expressed as the fraction of time above the MIC (*f*T_>MIC_), rather than concentration dependent (i.e. C_max_/MIC or AUC/MIC) [14]. Thus, no increased effect is expected for higher concentrations relative to the MIC. Whilst increasing concentrations may overcome drug resistance in some situations, specific alterations in drug target sites may be unaffected by increased concentrations [15]. Antibiotic stewardship through precise dosing to combat the propagation of antibiotic resistance should be considered, since this phenomenon is a growing concern in modern medicine. In addition, substantially lower doses per patient are more cost-efficient and could save money in the long term.

Also, it was investigated whether amoxicillin and cefazolin reached therapeutic concentrations in UCB. Most of these antibiotics too seem to exceed their corresponding MICs for GBS and/or *E.coli* [11] with the doses prescribed. The amoxicillin concentrations in UCB exceeded the MIC for GBS, however, were below the MIC for *E*. *coli* [11]. Since amoxicillin is prescribed as an alternative to benzylpenicillin for GBS prophylaxis in this context, the measured concentrations are satisfactory. For cefazolin, no target MIC for GBS could be defined [10, 11]. Nonetheless, cefazolin concentrations exceeded the MIC for *E. coli*. Since intrapartum cefazolin is prescribed for prophylaxis of maternal indications during caesarian sections rather than GBS prophylaxis, it seems that there is an added benefit of neonatal protection from *E. coli*. Gentamicin concentrations were not evaluated with respect to MIC, since gentamicin bactericidal effect is related to C_max_/MIC and concentrations in UCB did not represent C_max_. Gentamicin concentrations were above 1 mg/L in two of the three measurements in UCB. Such concentrations should be considered to prevent errors in therapeutic drug monitoring during subsequent gentamicin dosing of the neonate. It should be noted however that only 3 gentamicin concentrations were measured in UCB so any conclusions would be underpowered.

This is the first study analyzing the PK results of the Dutch obstetric benzylpenicillin protocol for GBS prophylaxis by assessing both UCB and neonatal plasma samples [7]. To our best knowledge, no evidence is publicly available describing therapeutic benzylpenicillin concentration target attainment in fetal/neonatal plasma following an intrapartum starting dose of 2,000,000 IU followed by 1,000,000 IU every 4 h. The use of a the log-linear regression line enabled the extrapolation of benzylpenicillin concentrations in UCB with limited data.

Limitations to the study were the limited number of samples. This severely hampered the applicability of gold standard statistical approaches such as population PK analyses. In addition, the absence of maternal PK blood samples complicated the applicability of population PK modeling and though physiology-based pharmacokinetics (PBPK) was considered as a potential method to assess and simulate the PK of benzylpenicillin, this was complicated due to the absence of basic demographic maternal data, such as maternal weight.

## Conclusions

Benzylpenicillin was detectable in therapeutic concentrations in UCB and equal to neonatal plasma concentrations. The current Dutch dosing protocol for benzylpenicillin dosing for intrapartum GBS prophylaxis is adequate even though it recommends lower doses as compared to international literature. A future study, combining maternal, UCB and neonatal plasma benzylpenicillin concentrations in a PBPK model should be conducted to provide a detailed assessment of prophylactic intrapartum benzylpenicillin treatment to prevent EOS. It may be beneficial to revise international benzylpenicillin dose recommendations in GBS prophylaxis to reduce unnecessary drug overexposure.

## Data Availability

Study data will be made available to referees at submission and to readers promptly upon request without reservations.

## Abbreviations

AUC: Area Under the Curve
EDTA: Ethylene diamine tetraacetic acid
EOS: Early-onset neonatal sepsis
GBS: Group B Streptococcus
IQR: Interquartile Range
IU: International units
LC–MS/MS: Tandem liquid chromatography-mass spectrometry
MIC: Minimal Inhibitory Concentration
PK: Pharmacokinetics
QC: Quality control
UCB: Umbilical Cord Blood
